# Microtexturing of the Conductive PEDOT:PSS Polymer for Superhydrophobic Organic Electrochemical Transistors

**DOI:** 10.1155/2014/302694

**Published:** 2014-01-22

**Authors:** Francesco Gentile, Nicola Coppedè, Giuseppe Tarabella, Marco Villani, Davide Calestani, Patrizio Candeloro, Salvatore Iannotta, Enzo Di Fabrizio

**Affiliations:** ^1^Bio Nano Engineering and Technology for Medicine (BioNEM), University Magna Graecia of Catanzaro, 88100 Catanzaro, Italy; ^2^Istituto Italiano di Tecnologia, Via Morego 30, 16163 Genova, Italy; ^3^Institute of Materials for Electronics and Magnetism (IMEM), National Research Council (CNR), Parco Area delle Scienze 37/A, 43124 Parma, Italy; ^4^King Abdullah University of Science and Technology (KAUST), Thuwal 23955-6900, Saudi Arabia

## Abstract

Superhydrophobic surfaces are bioinspired, nanotechnology artifacts, which feature a reduced friction coefficient, whereby they can be used for a number of very practical applications including, on the medical side, the manipulation of biological solutions. In this work, we integrated superhydrophobic patterns with the conducting polymer PEDOT:PSS, one of the most used polymers in organic electronics because highly sensitive to ionized species in solution. In doing so, we combined geometry and materials science to obtain an advanced device where, on account of the superhydrophobicity of the system, the solutions of interest can be manipulated and, on account of the conductive PEDOT:PSS polymer, the charged molecules dispersed inside can be *quantitatively* measured. This original substrate preparation allowed to perform electrochemical measurements on ionized species in solution with decreasing concentration down to 10^−7^ molar. Moreover, it was demonstrated the ability of the device of realizing specific, combined time and space resolved analysis of the sample. Collectively, these results demonstrate how a tight, interweaving integration of different disciplines can provide realistic tools for the detection of pathologies. The scheme here introduced offers breakthrough capabilities that are expected to radically improve both the pace and the productivity of biomedical research, creating an access revolution.

## 1. Introduction

Noninvasive analysis in biological fluids is gaining increasing interest in the diagnosis and experimental assessment of diseases. As for an example, it is very well understood that malignancies can be identified based on their molecular expression profiles [1, 2]. Exclusive markers extracted from serum, other biological fluids, or any sample obtained through minimally invasive techniques, can be realistically employed to discriminate cancers or other pathologies. The possibility to realize innovative sensing devices to measure specific analytes without any pain in the patient is a novel frontier in health control. Real time monitoring systems with noninvasive approach are limited but represent a crucial improvement in the preventive control of the patient. Electrochemical sensing by organic conductive polymer offers high sensitivity, direct and continuous control of electrolytes in solution and a transistor configuration able to amplify weak biosignals. The main limitations of this and other similar approaches are the low selectivity of the detection. To surpass these limitations, conventional conductive polymer can be conveniently modified to incorporate details at the micro- and nanoscales. The transition from the micro to the nano-scale dimensional control on surface features translates into increases in information quality, quantity, and density [1, 3, 4]. This increase, in turn, would render the utilization of similar biodevices effective in the diagnosis of cancer or other pathologies.

**Figure 1:**
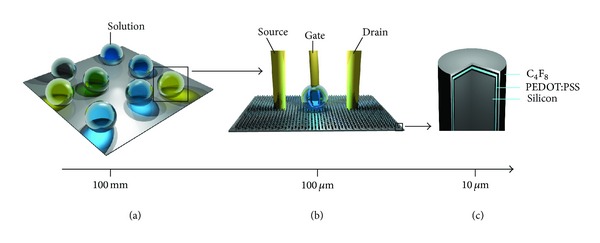
The device features hierarchically different scales in correspondence of which different functions are revealed. On a larger, millimetric scale the superhydrophobic substrate has the appearance of a continuous flat surface, where drops are repelled and solutions can be easily manipulated (a). On a mesoscale, a microstructure is revealed, that is the major attribute of the present biosensor device (b). The smallest unit of such a microstructure is the micropillar, comprising a silicon core and covered by a thin film of PEDOT:PSS and C_4_F_8_ polymer.

Organic thin film transistors (OTFTs) are widely explored as sensing devices, thanks to their peculiar properties, such as easiness of fabrication, low cost, flexibility, and biocompatibility. The most common OTFT is the organic field effect transistor (OFET), composed of an organic semiconductor film, contacted by the source-drain electrodes, and a gate dielectric (the insulator). Upon application of a gate voltage across the gate dielectric, an electrical double layer is formed at the channel interface and the current flowing between the source and drain can be modulated based on the field-effect doping. Organic electrochemical transistors (OECTs) are a second type of OTFT, in which the insulator layer, placed between the channel and gate electrode, is replaced with an electrolyte medium and the conductive polymer is electrochemically active. In the OECTs configuration the conductive polymer is in direct contact with the electrolyte, in which a gate electrode is immersed (Figure 1(b)). The overlapping of the polymer with the electrolyte defines the OECT channel. OECTs can be exploited as ion-to-electrons converters as well as biosensors: as sensing devices they have been so far used for the detection of a wide variety of chemical and biological analytes [5–7]. Their working mechanism is based on the doping/dedoping in the conducting polymer channel from ions through the electrolyte [8]. OECTs operate at low voltages, which makes them compatible with detection in aqueous environments and can be miniaturized and integrated in lab-on a chip configurations [9].

OECT active layer is commonly made of the conducting polymer poly(3,4-ethylenedioxythiophene):poly(styrenesulfonate) (PEDOT:PSS), which is a low cost, stable, and commercially available material. PEDOT:PSS can be processed into thin films from solution and has a high conductivity [10]. PEDOT is doped by PSS to increase the conductivity of the polymer and also to solve solubility problems [11]. Hence OECTs exploit electrolyte gating to achieve the transduction of ionic currents, whereby they are ideally suitable for sensing different chemo/biospecies dissolved in the electrolyte. As for some examples, OECTs have been successfully employed as wearable sensors for saline concentration in human sweat [12], where it was demonstrated how OECTs are able to reveal NaCl in the concentration range of human sweat (and also at 5 orders of magnitude lower in concentration) and could work in real time human sweat detection.

OECTs have been applied to detect lactate in aqueous solution [13] as well as the presence of glucose. Noticeably, those molecules are present in sweat, depending on their concentration in blood. OECT detection of metabolites [14, 15], micelles [16], neurotransmitters [17], cells [18], antibodies [19], and DNA [20] was also demonstrated. OECTs with single strand DNA probes on an Au gate electrode have been used to detect complementary DNA targets, where the sensing mechanism is related to the variation of the gate electrode potential induced by the DNA molecules on the gate surface. The device detects complementary DNA targets at concentrations as low as 1 nM and its detection limit is extended to 10 pM by pulse-enhanced hybridization of DNA [20].

The main limitations of this approach are the low selectivity of the sensing process, whereby different analytes with the same ion current charge would be erroneously considered like the same species. Moreover, the presence of interfering proteins, peptides, or extraneous ionized species in solution would blur the information content and the signal thereof. These limitations can be surpassed introducing an increasing degree of complexity into the geometry of the devices. On pattering the PEDOT polymeric films, one would obtain hierarchical structures with details at different scales. Those extra noncontinuous scales would provide the device with additional functions, including the capability of manipulating biological solutions.

Superhydrophobic surfaces (SHSs) are biomimetic, microstructured solids with a texture given by a regular hexagonal lattice of micropillars [21]. They deliver great potentials in biotechnology because they reveal superior properties compared to conventional flat surfaces, including extremely low friction coefficients [22–24]. On account of this, a millimetric drop would maintain a spherical shape if positioned upon those surfaces. Most importantly, during evaporation, the molecules contained in the drop can be concentrated, localized, or transported into specific points of the substrate [25]. This scheme has been used for a variety of applications. On integrating similar substrates with nano geometrical SERS devices, molecules have been detected in very low abundance ranges, with femto- to zepto-molar sensitivity [26, 27]; DNA fibers have been imaged thus gaining exclusive information of the double helix [28]; few, small Rhodamine molecules have been separated from large albumin proteins using nanoporous silicon as a molecular sieve [29, 30]. In general, a pattern at the micro- to nanoscales would allow to fragmentize, separate, or select the desired molecules from complex mixture. A correct design of the pattern can render this selection highly precise and reproducible. Therefore, on combining the OECT technology with SHSs, a new generation of devices can be obtained with the capability of sensing blends of proteins or other biomolecules, extracted from biological fluids, with high sensitivity, selectivity, and reliability.

In this work, we report on a novel microstructured, PEDOT:PSS-based biosensor. The sensor integrates an innovative material with nonconventional geometries. Specifically, an organic conductive layer (PEDOT:PSS) is shaped into a smart micropattern as in Figure 1. The geometry of the system would render this system superhydrophobic, with the capability of manipulating and controlling biological solutions of medical interest. This original substrate preparation allowed to perform electrochemical measurements on ionized species in solution (in this case, potassium chloride) with decreasing concentration down to 10^−7^ molar. Moreover, it was demonstrated the ability of the device of realizing specific, combined time and space resolved analysis of the sample, with elevated specificity and selectivity. Taken together, these results demonstrate how a tight, interweaving integration of multiple disciplines, including materials science and nanotechnology, can provide realistic tools for the detection of pathologies.

## 2. Materials and Methods

Superhydrophobic surfaces were realized. Those surfaces comprise micropillars disposed on the substrate to form a regular hexagonal motif; the pillars were therefore modified to incorporate a conductive PEDOT:PSS thin film. A fluorocarbon polymer (C_4_F_8_) was finally deposited on the devices which, on account of their hierarchical structures bridging different length scales [30], exhibit an increased hydrophobicity with contact angles as large as nearly 170°. Small drops (*V* < 10 *μ*L) of D.I. water containing infinitesimal amounts of analytes were gently positioned upon the surfaces. In experiments where the concentration of those analytes was varied over a significant range, the ion current of the species in solution was measured demonstrating a good sensitivity of the device. Deionized (DI) water was used for all experiments. All chemicals, unless mentioned elsewhere, were of analytical grade and were used as received.

### 2.1. Microfabrication of Patterned Superhydrophobic Surfaces

The patterned surfaces were characterized by periodic hexagonal tiling of cylindrical pillars. The diameter *d* and the distance *l* between the pillars were chosen in accordance with a criterion of optimal design, that is, *d* = 10 *μ*m and *l* = 20 *μ*m, while the height of the pillars (*h*) was 10 *μ*m. In doing so, a small fraction of solid *ϕ* = *π*/4 *d* ^2^/(*l*+*d*)^2^ ~ 0.09 was secured, assuring an increased hydrophobicity with contact angles approaching 170°, with *ϕ* still sufficiently large to prevent the collapse of the drop at the early stage of evaporation [25]. P-doped, (100) silicon wafers with resistivity of 5–10 Ohm/cm were used as substrates; they were cleaned with acetone and isopropanol to remove possible contaminants and then etched with a 4% hydrofluoric acid (HF) solution. The wafers were then rinsed with DI water and dried with N_2_. Standard optical lithography techniques (Karl Suss Mask Aligner MA 45, Suss MicroTec GA, Garching, Germany) were employed to generate regular patterns of disks within a layer of negative tone resist (AZ5214) that was spin-coated onto clean silicon wafers. The masks necessary for optical lithography were fabricated using standard Electron Beam Lithography (Crestec CABL-9000C Electron Beam Lithography system) methods. The disks served as a mask in a deep reactive ion etching process (DRIE) (MESC Multiplex ICP, STS, Imperial Park, Newport, UK), whereby the final structures were obtained in the form of cylindrical pillars with an aspect ratio greater than 2. The DRIE process utilized was a pulsed, time-multiplexed etching that alternates repeatedly between two modes and namely (i) an isotropic plasma etch, where SF_6_ is used, and (ii) a deposition of a chemically inert passivation layer that is C_4_F_8_, as described in [31]. On removing the residual resist with piranha solution (H_2_SO_4_ : H_2_O_2_ = 3 : 1 v/v), the substrates were therefore prepared for the formation of the conductive polymeric PEDOT:PSS thin film, that was spin-coated on the sample at 1500 rpm for 30 s. The substrates, as a whole, were then covered with a thin (few nm) film of a teflon-like (C_4_F_8_) polymer to ensure hydrophobicity; to do this, a modified Bosch/RIE process was utilized, where solely the passivation mode was activated. In this phase, all gas flows, including SF_6_, argon, and oxygen, are set to zero, with the exception of the chemical inert passivation layer C_4_F_8_.

### 2.2. The Conductive PEDOT:PSS Thin Film

A solution of poly(3,4-ethylenedioxythiophene) doped with poly(styrene sulfonate), PEDOT:PSS (H.C. StarckClevios PH500), was spun onto the silicon substrate with pillars substrate so that an estimated film thickness *d* ~ 80 nm was achieved. The PEDOT:PSS solution was previously doped with ethylene glycol (Sigma Aldrich) to enhance its electrical conductivity and dodecyl benzene sulfonic acid (DBSA) surfactant (Sigma Aldrich) to improve film forming. After the spinning the device was baked on a hotplate at 140°C for 60 min. Silver metal contacts over the conductive PEDOT:PSS film were realized at a distance of ~10 mm to be used as source and drain contacts.

### 2.3. SEM Characterization

Several SEM images of the samples were captured to assure reproducibility and repeatability, utilizing a Dual Beam (SEM-FIB) - FEI Nova 600 NanoLab system. During the acquisitions beam energies of 5 and 15 keV, and corresponding electron currents of 0.98 pA and 0.14 nA, were used. In some cases the mode 2 configuration was set, through which images can be magnified over 2500 × 10^3^ times, and ultrahigh resolution can be achieved.

### 2.4. Surface Contact Angle Measurement

Surface hydrophobicity of the samples was determined by measuring the water contact angle with one drop (5 mL) of deionized water using an automatic contact angle meter (KSV CAM 101, KSV Instruments LTD, Helsinki, Finland) at room temperature. Several measurements were performed on each substrate to evaluate the average contact angle *θ*, at 5 s. Following the Young-Dupre equation, the energy of adhesion *g* per unit area at the substrate/water interface was defined as *γ* = *γ*_LG_(1 + cos⁡*θ*), where *γ*_LG_ is the air/water surface tension (72.8 mJ/m^2^ at 20°C).

### 2.5. Measuring the Electric Characteristic of Ionized Species in Solution

The electrical response of OECT has been measured using a 2-channel source/measure precision unit (Agilent B2902A), controlled by a home-made LabView software. Source and drain electrodes were realized on the PEDOT:PSS by Ag metal contacts. A drop of electrolyte has been suspended over the pillars, between the two contacts on the surface, while Ag wire, immersed into the electrolyte drop, acted as gate electrode (Figure 3(a)). OECT devices were characterized by recording output characteristics (*I*_ds_ versus *V*_ds_) of KCl at concentration of 1 × 10^−9^ M (Figure 3). The output curves as in Figure 3(b) have been acquired by spanning *V*_ds_ between 0 and −1 V, in steps of 0.1 V, for a fixed *V*_gs_ varied in the range between 0 and 1 V, with steps of 0.2 V. OECT measurements were acquired by measuring *I*_ds_ versus time (Figure 3(c)) under a constant *V*_ds_ = −0.4 V, while pulsing *V*_gs_ between 0 V and a positive value that was gradually increased from 0 V to 1 V with a step of 0.2 V. The gate current (*I*_gs_) was simultaneously acquired during measurements (Figure 3(d)). Then transfer curves shown in Figure 4 have been acquired, fixing *V*_ds_ = −0.4 V and ranging *V*_gs_ between 0 and 1 V, with steps of 0.2 V. *I*_ds_ was acquired as a function of time for 60 s, at each step, a time long enough to achieve a nearly steady drain current value after the *V*_gs_ value was varied. Different concentration of KCl was analyzed from 10^−7^ to 10^−1^ M. The application of a drain voltage *V*_ds_ induces hole drifting in the channel, generating the channel current *I*_ds_. When a positive gate voltage (*V*_gs_) is applied, cations (M^+^) from the electrolyte enter the PEDOT:PSS channel and dedope it according to (1) as follows:

(1) $$\begin{array}{c} {\text{PEDOT}}^{+}\text{:}\;{\text{PSS}}^{-}+{\text{M}}^{+}+{\text{e}}^{-}⟷{\text{PEDOT}}^{0}+{\text{M}}^{+}\text{:}\;{\text{PSS}}^{-} \end{array}$$

We refer to this as to a dedoping process, because cations entering the polymer make the drain current module |*I*_ds_| decrease due to the smaller number of holes available for conduction. On the contrary, when *V*_gs_ = 0 V is applied, ion diffusion occurs from the PEDOT:PSS to the electrolyte, thus increasing the number of conducting holes. This is named a doping process, as in this case |*I*_ds_| increases. As a consequence, the drain current module |*I*_ds_| decreases when *V*_gs_ > 0 V is applied and increases when *V*_gs_ is set again to 0 V (Figure 3(b)). OECT current response is expressed as current modulation Δ*I*/*I*_0_ = [(*I* − *I*_0_)/*I*_0_], where *I* is the drain current value measured for *V*_gs_ > 0 V and *I*_0_ is the *I*_ds_ value for *V*_gs_ = 0 V. For each measurement, *I* and *I*_0_ values were obtained from current transient measurements considering the quasi-steady state current values.

### 2.6. Statistical Analysis

All the data are reported as the sample mean ± the  standard deviation (SD). The ionic currents presented in Figure 4 are averaged over 10 measurements repeated for 6 different samples. In estimating the error bars, the conventional definition of standard deviation is utilized. Pair-wise comparisons between means of different groups were performed using a Student's *t*-test (two tailed, unpaired) where, for each couple of normally distributed populations, the null hypothesis that the means are equal was verified. Everywhere in the text the difference between two subsets of data is considered statistically significant if the Student's *t*-test gives a significant level *P* (*P* value) less than 0.05. In some cases, the *P* value can be well below than 0.05, as reported in the text where necessary.

## 3. Results

### 3.1. The Micropatterned Devices

**Figure 2:**
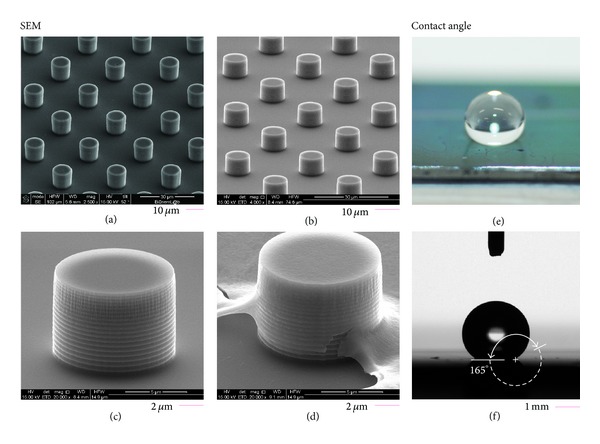
SEM images of the micropillars composing the superhydrophobic device; the pillars are arrayed to form regular geometries and these cover regions as large as several hundreds of microns without defects or imperfections ((a), (b)). Larger magnifications reveal, at smaller scales, the conductive PEDOT:PSS thin film ((c), (d)). A fluorocarbon polymer (C_4_F_8_) is finally deposited on the devices which, on account of their hierarchical structures bridging different length scales [30], exhibit an increased hydrophobicity with contact angles as large as 165° ((e), (f)).

Several SEM micrographs of the superhydrophobic devices were taken over different samples to assess uniformity and reproducibility. In Figures 2(a) and 2(b), silicon pillars are arrayed over large square areas sizing up to some hundreds of micrometers per side, with minor or no defects in the structures which thus recover a perfect hexagonal lattice. This would verify the fabrication process capability to attain extreme control over the key characteristics of the micropillars such as shape and size, at least on a large scale. On a smaller scale, as in Figure 2(c), one can notice the exposed top surface of the pillars covered by the PEDOT:PSS polymeric conductive thin film. This is even more evident in Figure 2(d), where a defect in the structure reveals the PEDOT:PSS membrane which drapes the silicon micropillar, yet not altering its geometry. A fluorocarbon polymer, conveniently deposited on the devices as described in the Materials and Methods, would endow to the systema multiscale, hierarchical structure. This structure, on bridging the micro- and nanoscales [30, 32, 33], induces an artificial, increased hydrophobicity with contact angles, for this particular configuration, as large as 165° (Figures 2(e)–2(f)). Photographs of the real device are reported in a separate Supporting Information file (see Supplementary Materials available online at http://dx.doi.org/10.1155/2014/302694).

### 3.2. Testing of the Devices as Biosensors

**Figure 3:**
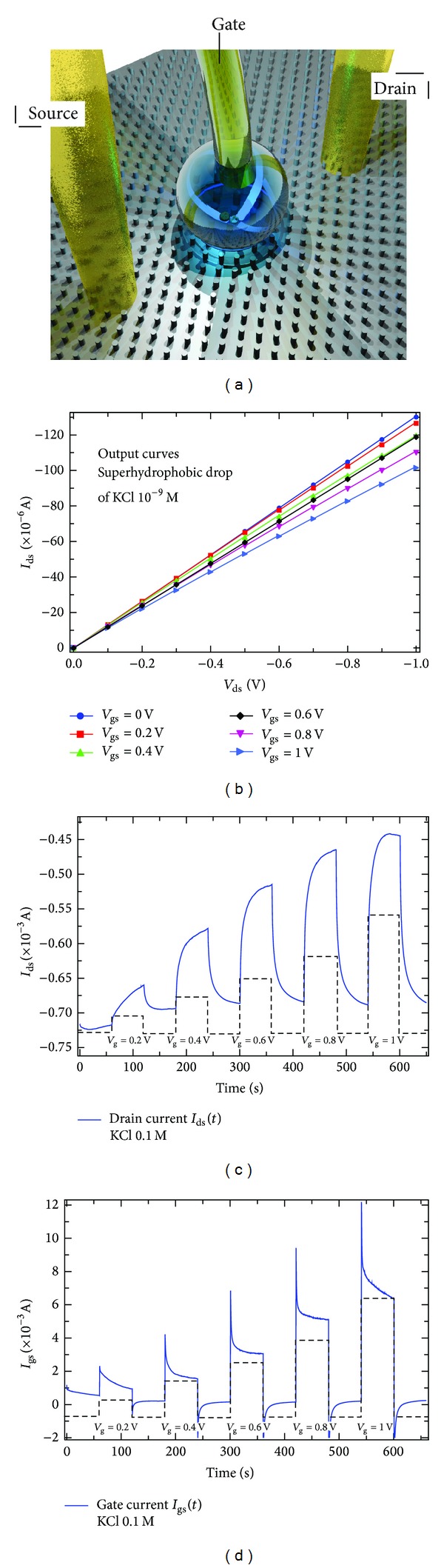
The devices have been tested using a solution of KCl in water to evaluate their electric characteristics and their sensing performances. In (a) the sketch of the device architecture is reported. Two electrodes, source and drain, are connected to the PEDOT:PSS on the substrate surface. Between the two metal electrodes a drop of electrolyte solution is suspended over the pillar surface with a gate silver electrode on the top. In (b), the transfer characteristic of the transistor at 10^−9^ M is reported. The transfer characteristic shows the working characteristics of the OECT on the superhydrophobic surface. In (c), the measurement of *I*_ds_ current in function of time for increasing gate voltages for KCl at 0.1 M is reported. In (d), the current between gate and source in function of time during the same increasing gate voltages is instead reported.

The devices have been tested using different saline solutions, KCl, NaCl, MgCl, ZnCl, at different concentrations (from 10^−7^ to 10^−3^ M) in water to evaluate their electric characteristics and their sensing performances. In Figure 3(a) the sketch of the device architecture is reported. Two electrodes, source and drain are connected to the PEDOT:PSS on the substrate surface. Between the two metal electrodes a drop of electrolyte solution is suspended over the pillar surface with a gate silver electrode on the top.

**Figure 4:**
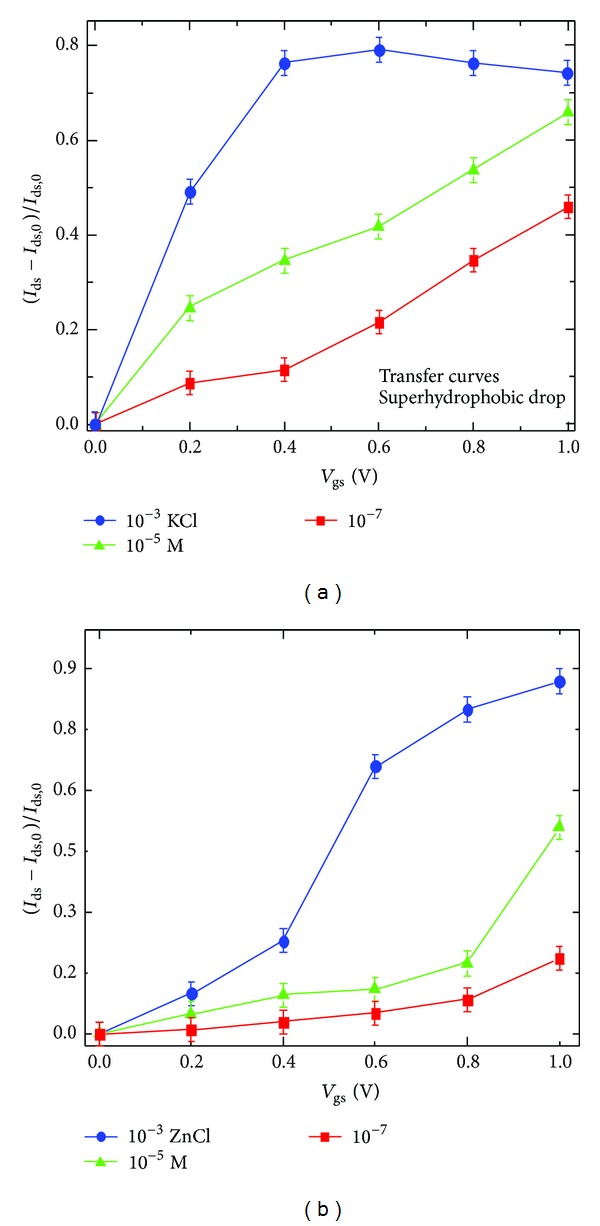
The response of the device to different KCl (a) and ZnCl (b) concentrations, ranging from 10^−7^ to 10^−3^ M, was reported as the current modulation Δ*I*/*I*_0_ = |*I*_ds,*off*⁡_ − *I*_ds,0_|/*I*_ds,0_. The reported values of modulation depend on the concentration of KCl. Notice how the traces correctly increase for increasing values of KCl concentration. Also notice that the differences between different groups of means, as reported in the figure, are statistically significant for all the considered values of applied voltage.

In Figure 3(b) the transfer characteristic (*I*_ds_ versus *V*_gs_) of the transistor with a KCl solution at 10^−9^ M on the superhydrophobic surface is shown. The different slope and the separation for the *I*_ds_ curves at different gate voltages show an effective working regime for the electrochemical transistor with a suspended drop on the pillar structure. In Figure 3(c) the effective sensing of KCl at 0.1 M is shown. In these measurements *I*_ds_ is reported as a function of time, while different *V*_gs_ are applied to the electrolyte drop *via* the gate electrode. A series of increasing voltages from 0.2 to 1 V has been applied for 60 s, alternated to 0 V, while a fixed voltage of −0.4 V is applied between source and drain. The fixed voltage allows to measure a current in the PEDOT:PSS, which is quite constant when a 0 V voltage is on the gate. This current value represents the baseline of the sensing measurement. When positive voltage is applied to the gate, they act on the ions dispersed in the solution, in this case K^+^, and they force the ions to move in the direction of the PEDOT:PSS on the pillar top. Hence, applying a positive voltage, the K^+^ penetrate in the PEDOT:PSS film, reducing its conductivity, as shown for each value of the potential in the rising edge of the curve, which reaches rapidly a saturation, as all the ions are forced in the film and dedope it. When the voltage is turned to 0, the ions start a diffusion process back in the electrolyte, improving the conductivity to the previous baseline level. The reduction of conductivity is proportional to the intensity of the applied voltage and depends on the K^+^ concentration in the solution. In Figure 3(d) it is reported the current between gate and source as a function of time, during the same series of measurements of Figure 3(c). The *I*_gs_ current is related to the redox reaction between Cl^−^ ions and the Ag gate electrode. At the surface of the Ag electrode Cl^−^ ions react with Ag, forming AgCl and exchanging electrons. This working condition for OECT is called Faradaic regime and corresponds to a significant gate current which is comparable with the *I*_ds_; this is a typical regime for the saline solution and Ag gate electrode. The steady-state current due to reduction/oxidation redox reactions is sustained in the electrolyte. As a consequence, the OECT gate-source current *I*_gs_, which results from capacitive effects and/or from redox reactions at the gate electrode, can be used as a fingerprint of the regime of OECT operation. Both Figures 3(c) and 3(d) show that the OECT realized on the superhydrophobic platform has perfectly worked as a sensible sensing device for KCl with an appropriate response time and effective recovery of the baseline level. The superficial treatment with C_4_F_8_, used to improve the hydrophobicity of the surface, does not prevent the interaction of PEDOT:PSS with the solution in the drop, as could be seen by the modulation due to the iteration of the K^+^. It is probably due to an incomplete and porous functionalization of the PEDOT:PSS surface by C_4_F_8_, which let the ions pass through the hydrophobic layer. In Figure 4(a), the response for different KCl concentration, from 10^−7^ to 10^−3^ M, has been reported. The response of the device is expressed as the current modulation Δ*I*/*I*_0_ = |*I*_ds,*off*⁡_ − *I*_ds,0_|/*I*_ds,0_, where *I*_ds,*off*⁡_ is the off current (*V*_gs_ > 0) and *I*_ds,0_ the on current (*V*_gs_ = 0). The reported values of modulation depend on the KCl concentration for different *V*_g_. The red line, representing 10^−7^ M, produces a modulation in current that is lower for each *V*_g_ value respect to the green line of 10^−5^ M, and coherently 10^−3^ results with a higher response with respect to 10^−5^ M. The differences between the different groups of means, as presented in Figure 4(a), are statistically significant for all the considered values of voltage. The dependence on the concentration at a fixed voltage allows to use the device as sensitive sensor for saline concentration in water. The application of an appropriate gate voltage, like 0.6 V for example, presents a clear difference of modulation for each concentration of KCl, which demonstrates the sensing applicability for the device. The modulation response of ZnCl is reported in Figure 4(b). Similarly to the behavior of KCL, zinc chloride demonstrates a dependence of the modulation amplitude to the saline concentration and a high sensitivity to the concentration of Zn in solution, starting from 10^−7^ M. This modulation comparison assesses uniformity and reproducibility of the measurement for different species (NaCl, KCl, MgCl, and ZnCl). The modulation response of MgCl is reported in a separate Supporting Information file.

### 3.3. Measuring Different Species in a Solution

**Figure 5:**
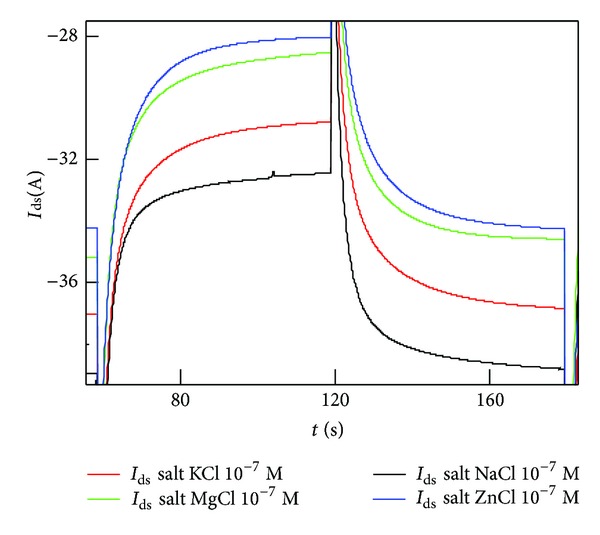
Transient response of the device (*I*_ds_ in function of time at the switching of 0.2 V of gate voltage) for different species in a solution that are magnesium (Mg^++^), zinc (Zn^++^), potassium (K^+^), and sodium (Na^+^). The transient behavior for Zn and Mg is similar in shape and this is easily accepted considering that zinc and magnesium have physical constants that are comparable. In the same diagram, differently from the case of Zn and Mg, and with a lower intensity, the ionic current of potassium and sodium is reported.

In Figure 5, the transient behavior of the device is analyzed for different species in solution that are magnesium (Mg^++^), zinc (Zn^++^), sodium (Na^+^), and potassium (K^+^), with a similar *c* = 10^−3^ M concentration. These elements feature different diffusion coefficients, ranging from *D* = 0.706 × 10^−9^ m^2^/s and *D* = 0.703 × 10^−9^ m^2^/s for magnesium and zinc to *D* = 1.334 × 10^−9^ m^2^/s and *D* = 1.96 × 10^−9^ m^2^/s, for sodium and potassium. Moreover, the charge number of those diffusing species is also different, being *z* = 1 for sodium and potassium and *z* = 2 for magnesium and zinc. Therefore, the described species feature different chemical and physical properties. On observing the experimental current sensed by the device as a function of time (Figure 5), one can notice that those chemical and physical differences are reflected by the characteristic time response of the salts in analysis. The transient behavior for Zn and Mg is similar in shape and this is easily accepted considering that zinc and magnesium have physical constants that are comparable. In the same diagram, differently from the case of Zn and Mg, and with a lower intensity, the ionic current of potassium is reported. Notice that Mg, Zn, K, and Na are ordered for decreasing intensity in Figure 5. This is consistent with the fact that Mg and Zn have a charge number *z* = 2, and present a comparable diffusion coefficient, while the different behaviors of K and Na in modulation dynamic, with a lower evolution in time, could be related to their *z* = 1 charge number. Among K and Na a different kinetic behavior of the transient corresponds to a different diffusion coefficient: K presents higher transient, corresponding to a higher diffusion coefficient (*D* = 1.96 × 10^−9^ m^2^/s), while Na (*D* = 1.334 × 10^−9^ m^2^/s), which presents lower intensity transient, has a lower diffusion coefficient. This specific hint could be used as selective information in the detection process of the device, that could be enhanced and controlled by the micropatterned structure of the superhydrophobic surface, which is able to control and separate different species with different diffusion coefficients.

This is better explained considering that the output of measurement would depend on the transportation of ions in solution. The mechanisms of ion transportation in the electrolyte would depend, in turn, on the externally applied voltage and on the electrical mobility of those ions in the solution; that is described by the well-known Einstein-Smoluchowski relation *m* = *q*/*KTD*. For a fixed temperature, the combined effect of the charge *q* and the diffusion *D* would therefore have an effective impact on the output of the sensor. Considering that the Brownian coefficient of diffusion is inversely related to the hydrodynamic diameter of a diffusing molecule, different species in a solution, with different sizes and thus mobilities, would behave differently upon the application of the same stimulus, and this is the case of the species considered here for the analysis. This scheme delivers the ability to sense those differences; therefore, it can be utilized for detecting and recognizing specific species in a solution with an elevated sensitivity and selectivity.

## 4. Discussions and Conclusions

Despite the name, superhydrophobic surfaces are in reality 2 + 1 dimensional objects, where the +1 dimension is the pattern, at the micro- or nanoscale, that decorates those surfaces. The presence of this pattern gives to the system certain attributes and advantages with respect to conventional devices that can be recapitulated as follows [24]: (i) the geometry and positioning of liquid droplets can be easily controlled; (ii) micropatterns can be prefilled with aqueous solutions without the need for surfactants; (iii) droplets can be positioned extremely close to each other on a surface; (iv) superhydrophobic regions can be used to create patterns to control bio-adhesion; (iv) the discontinuous dewetting effect arising from the extreme difference in contact angle hysteresis between superhydrophilic and superhydrophobic regions can be used to passively dispense aqueous solution into the super-hydrophilic spots without wetting the superhydrophobic background. Collectively, these properties are of interest for the very large community of researchers working in the field of biomedical devices and molecular diagnostics, where the most severe limitation of current approaches is the difficulty of handling extremely low concentrated solutions containing few molecules. Micro- to nanopatterned systems offer realistic possibilities to overcome these limitations.

In this work, we integrated similar superhydrophobic patterns with the conducting polymer PEDOT:PSS that is a material that permits the electrical characterization of ionized species in a solution. In doing so, we combined geometry and materials science to obtain advanced devices where, on account of the superhydrophobicity of the system, the solutions of interest can be manipulated and, on account of the conductive PEDOT polymer, the charged molecules dispersed inside can be *quantitatively* measured. Specifically, in experiments where the concentration of potassium chloride and other salts was varied over a significant range, we demonstrated a very high sensitivity of the device to the number of ions in solution. Moreover, in experiments where different species in a solution were measured, we demonstrated a very high sensitivity of the response of the system to the physical and chemical properties of those species, including the mass of the diffusing molecules. This demonstrates the selectivity abilities of the device.

**Figure 6:**
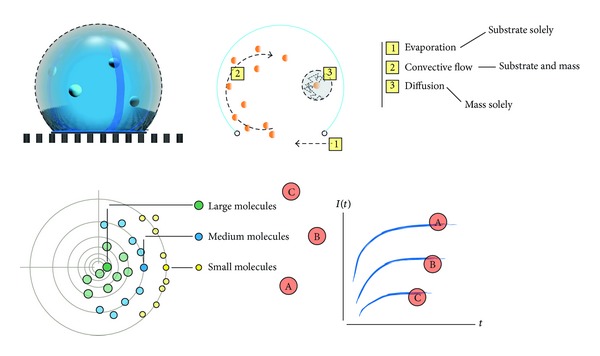
The cartoon is a representation of a textured, superhydrophobic conductive polymer, where a millimetric drop would maintain a spherical shape, with convective flows herein generated due to the celebrated Marangoni effect. These flows of masses depend on the size of the drop and on its curvature, that is, on the angle of contact on the surface. Consequently, they may be controlled on controlling the geometry of the substrate. These extra noncontinuous scales are responsible for the velocity vector field in the solution domain. The displacement of a molecule in a solution would thus depend on the interplay of diffusion, convective flow, and evaporation. Controlling the relative contribution of these mechanisms to the displacement of a molecule would allow to separate different species on a quantitative basis.

Interestingly, *selectivity* is a property that may be improved on micro texturing the conductive PEDOT:PSS polymer, and this is explained in the following. The measurement of a species in a solution using an OECT device, that is, the response of the system, results from a sequence of tightly interweaving processes, including the transport of the species from the electrolyte into the conductive polymer. In the Results (Figure 5), we demonstrate that the transport of the species in the electrolyte determines the final response of the system. In conventional OECT devices, the mechanisms of ion transportation in the electrolyte are driven by the externally applied electric field and by pure diffusion. Differently from this, in a textured, superhydrophobic conductive polymer, a millimetric drop would maintain a spherical shape, where convective flows are generated due to the celebrated Marangoni effect (Figure 6). These flows of masses depend on the size of the drop and on its curvature, that is, on the angle of contact on the surface. Consequently, they may be controlled on controlling the geometry of the substrate. These extra noncontinuous scales are responsible for the velocity vector field in the solution domain, that is, an additional convective term, and the evaporation of the solute over time. The displacement of a molecule in a solution would thus depend on the interplay of diffusion, convective flow, and evaporation. Nanotechnologies provide a tool to fabricate the substrate on the basis of a specific design to obtain a convective flux and an evaporation dynamics with the desired characteristics. On changing the relative contribution of convection, evaporation, and diffusion, on the motion of molecules, one would obtain different, mass resolved, system responses. Tuning the geometry of the substrate would thus unbalance the system to obtain a specific response. Considering that the motion of molecules under diffusion and convection is related to the hydrodynamic diameter of a molecule, different species in a solution, with different sizes, would behave differently upon the application of the same stimulus. This scheme, and its more sophisticated evolution that will be developed upon time, can be utilized for detecting and recognizing specific species in a solution.

Considering all this, the device introduced in the present paper is a *proof of principle* whose purpose is verifying that the described method has the potentials of being used for a variety of applications of biomedical interest, including the diagnosis of diseases starting from serum, other biological fluids, or any sample obtained through minimally or noninvasive techniques and the monitoring and control of the ongoing physiological conditions of individuals. Moreover, the integration of nanotopography with such a conductive polymer may serve to investigate specific biological functions at the nano scales, comprising the adhesion, growth, and differentiation of stem cells [34] or the formation of intricate cell complexes [35, 36]. For all this, the scheme here introduced offers breakthrough capabilities that are expected to radically improve both the pace and the productivity of biomedical research, creating an access revolution.

## Supplementary Material

A separated supplementary information file is available on line. In the document, (Supporting Information #1) photographs of the real micro patterned OECT devices are reported, and (Supporting Information #2) the current modulation measured by the OECT device is shown as a function of the voltage at the gate for a varying concentration (from 10^−7^ to 10^−3^ M) of the target species (MgCl).

## Abbreviations

SEM: Scanning electron microscope
SH: Superhydrophobic
OECT: Organic electrochemical transistors
PEDOT:PSS: Poly(3,4-ethylenedioxythiophene):poly(styrenesulfonate).

## References

[1] Ferrari M (2005). Cancer nanotechnology: opportunities and challenges. *Nature Reviews Cancer*.

[2] Posadas EM, Simpkins F, Liotta LA, MacDonald C, Kohn EC (2005). Proteomic analysis for the early detection and rational treatment of cancer—realistic hope?. *Annals of Oncology*.

[3] Helmus MN (2006). How to commercialize nanotechnology. *Nature nanotechnology*.

[4] Whitesides GM (2003). The “right” size in nanobiotechnology. *Nature Biotechnology*.

[5] Mabeck JT, Malliaras GG (2006). Chemical and biological sensors based on organic thin-film transistors. *Analytical and Bioanalytical Chemistry*.

[6] Tarabella G, Mohammadi FM, Coppedè N (2013). New opportunities for organic electronics and bioelectronics: ions in action. *Chemical Science*.

[7] Lin P, Yan F (2012). Organic thin-film transistors for chemical and biological sensing. *Advanced Materials*.

[8] Bernards DA, Malliaras GG (2007). Steady-state and transient behavior of organic electrochemical transistors. *Advanced Functional Materials*.

[9] Mabeck JT, Defranco JA, Bernards DA, Malliaras GG, Hocdé S, Chase CJ (2005). Microfluidic gating of an organic electrochemical transistor. *Applied Physics Letters*.

[10] Cicoira F, Sessolo M, Yaghmazadeh O, Defranco JA, Yang SY, Malliaras GC (2010). Influence of device geometry on sensor characteristics of planar Organic electrochemical transistors. *Advanced Materials*.

[11] Tarabella G, Santato C, Yang SY, Iannotta S, Malliaras GG, Cicoira F (2010). Effect of the gate electrode on the response of organic electrochemical transistors. *Applied Physics Letters*.

[12] Tarabella G, Villani M, Calestani D (2012). A single cotton fiber organic electrochemical transistor for liquid electrolyte saline sensing. *Journal of Materials Chemistry*.

[13] Khodagholy D, Curto VF, Fraser KJ (2012). Organic electrochemical transistor incorporating an ionogel as a solid state electrolyte for lactate sensing. *Journal of Materials Chemistry*.

[14] Bernards DA, MacAya DJ, Nikolou M, Defranco JA, Takamatsu S, Malliaras GG (2008). Enzymatic sensing with organic electrochemical transistors. *Journal of Materials Chemistry*.

[15] Yang SY, DeFranco JA, Sylvester YA (2009). Integration of a surface-directed microfluidic system with an organic electrochemical transistor array for multi-analyte biosensors. *Lab on a Chip - Miniaturisation for Chemistry and Biology*.

[16] Tarabella G, Nand G, Villani M Organic electrochemical transistors monitoring micelle formation. *Chemical Science*.

[17] Yang SY, Kim BN, Zakhidov AA (2011). Detection of transmitter release from single living cells using conducting polymer microelectrodes. *Advanced Materials*.

[18] Lin P, Yan F, Yu J, Chan HLW, Yangù M (2010). The application of organic electrochemical transistors in cell-based biosensors. *Advanced Materials*.

[19] Kim D-J, Lee N-E, Park J-S, Park I-J, Kim J-G, Cho HJ (2010). Organic electrochemical transistor based immunosensor for prostate specific antigen (PSA) detection using gold nanoparticles for signal amplification. *Biosensors and Bioelectronics*.

[20] Lin P, Luo X, Hsing I-M, Yan F (2011). Organic electrochemical transistors integrated in flexible microfluidic systems and used for label-free DNA sensing. *Advanced Materials*.

[21] Blossey R (2003). Self-cleaning surfaces—virtual realities. *Nature Materials*.

[22] Lafuma A, Quéré D (2003). Superhydrophobic states. *Nature Materials*.

[23] McHale G, Shirtcliffe NJ, Newton MI (2004). Super-hydrophobic and super-wetting surfaces: analytical potential?. *Analyst*.

[24] Ueda E, Levkin PA (2013). Emerging applications of superhydrophilic-superhydrophobic micropatterns. *Advanced Materials*.

[25] Gentile F, Coluccio M, Coppedè N (2012). Superhydrophobic surfaces as smart platforms for the analysis of diluted biological solutions. *ACS Applied Materials and Interfaces*.

[26] De Angelis F, Gentile F, Mecarini F (2011). Breaking the diffusion limit with super-hydrophobic delivery of molecules to plasmonic nanofocusing SERS structures. *Nature Photonics*.

[27] Gentile F, Das G, Coluccio ML (2010). Ultra low concentrated molecular detection using super hydrophobic surface based biophotonic devices. *Microelectronic Engineering*.

[28] Gentile F, Moretti M, Limongi T (2012). Direct imaging of DNA fibers: the visage of double helix. *Nano Letters*.

[29] Gentile F, Coluccio ML, Accardo A (2011). Nanoporous-micropatterned-superhydrophobic surfaces as harvesting agents for few low molecular weight molecules. *Microelectronic Engineering*.

[30] Gentile F, Battista E, Accardo A (2011). Fractal structure can explain the increased hydrophobicity of nanoporous silicon films. *Microelectronic Engineering*.

[31] Limongi T, Cesca F, Gentile F (2013). Nanostructured superhydrophobic substrates trigger the development of 3D neuronal networks. *Small*.

[32] Nosonovsky M, Bhushan B (2007). Hierarchical roughness optimization for biomimetic superhydrophobic surfaces. *Ultramicroscopy*.

[33] Nosonovsky M, Bhushan B (2007). Biomimetic superhydrophobic surfaces: multiscale approach. *Nano Letters*.

[34] Chen W, Villa-Diaz LG, Sun Y (2012). Nanotopography influences adhesion, spreading, and self-renewal of human embryonic stem cells. *ACS Nano*.

[35] Bettinger CJ, Langer R, Borenstein JT (2009). Engineering substrate topography at the Micro- and nanoscale to control cell function. *Angewandte Chemie—International Edition*.

[36] Gentile F, Tirinato L, Battista E (2010). Cells preferentially grow on rough substrates. *Biomaterials*.

