# Supplementary material for: Microtexturing of the Conductive PEDOT:PSS Polymer for Superhydrophobic Organic Electrochemical Transistors
Source: Biomed Res Int. 2014 Jan 22;2014:302694. doi: 10.1155/2014/302694 (PMC3919119; doi:10.1155/2014/302694)
Supplement: Supplementary file 1 — A separated supplementary information file is available on line. In the document, (Supporting Information #1) photographs of the real micro patterned OECT devices are reported, and (Supporting Information #2) the current modulation measured by the OECT device is shown as a function of the voltage at the gate for a varying concentration (from 10−7 to 10−3 M) of the target species (MgCl). [file 302694.f1.pdf]

Micro-texturing of the conductive PEDOT:PSS polymer for super-hydrophobic Organic Electrochemical Transistors

Francesco Gentile[1,2,†,*], Nicola Coppedè[3,†], Giuseppe Tarabella[3], Marco Villani[3], Davide Calestani[3], Patrizio Candeloro[1], Salvatore Iannotta[3], Enzo Di Fabrizio[4,1]

[1]BioNEM (Bio Nano Engineering and Technology for Medicine), University Magna Graecia of Catanzaro - 88100, Italy

[2]Istituto Italiano di Tecnologia, Via Morego 30, 16163 Genova, Italy

[3]IMEM-CNR Parco Area delle Scienze 37/A - 43124 Parma, Italy

[4]King Abdullah University of Science and Technology (KAUST), Thuwal 23955-6900, Kingdom of Saudi Arabia

Supporting Information #1

Here, photographs of the real micro patterned OECT devices are reported. Notice, in Figures from Figure S1.1 to S1.3, the solution assuming a neat spherical shape with contact angles larger than 170°. Also notice the source and drain electrodes integrated into the micro-patterned substrate, while the gate is immerged in the solution.

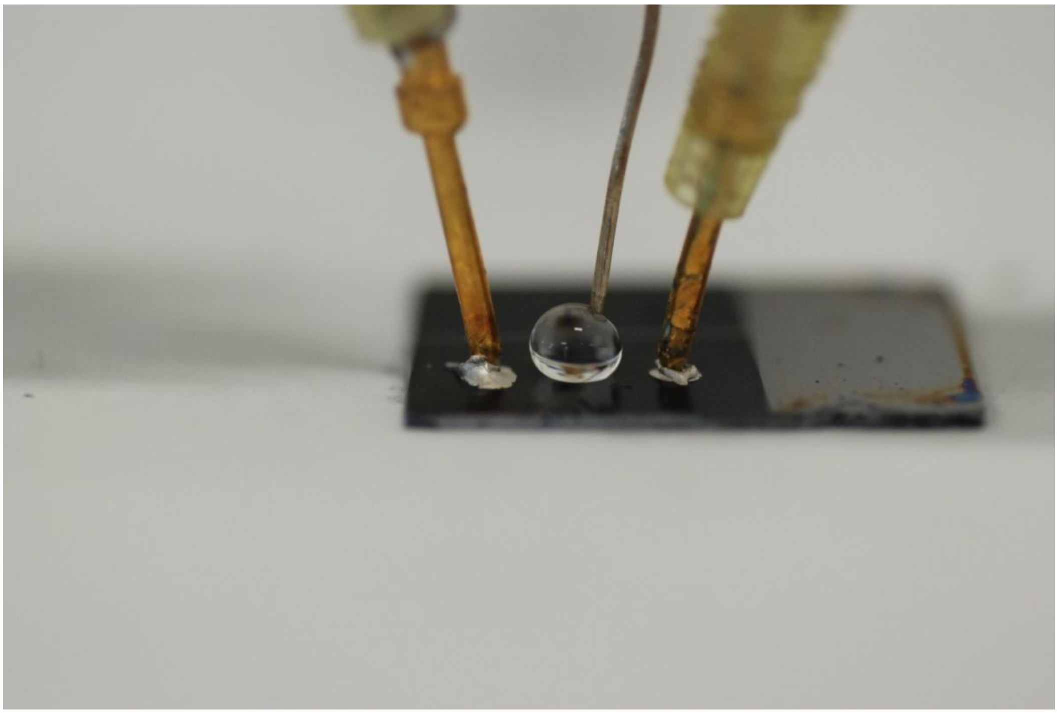

Figure S1.1

[†]These authors contributed equally to this work
[*]Corresponding author: gentile@unicz.it

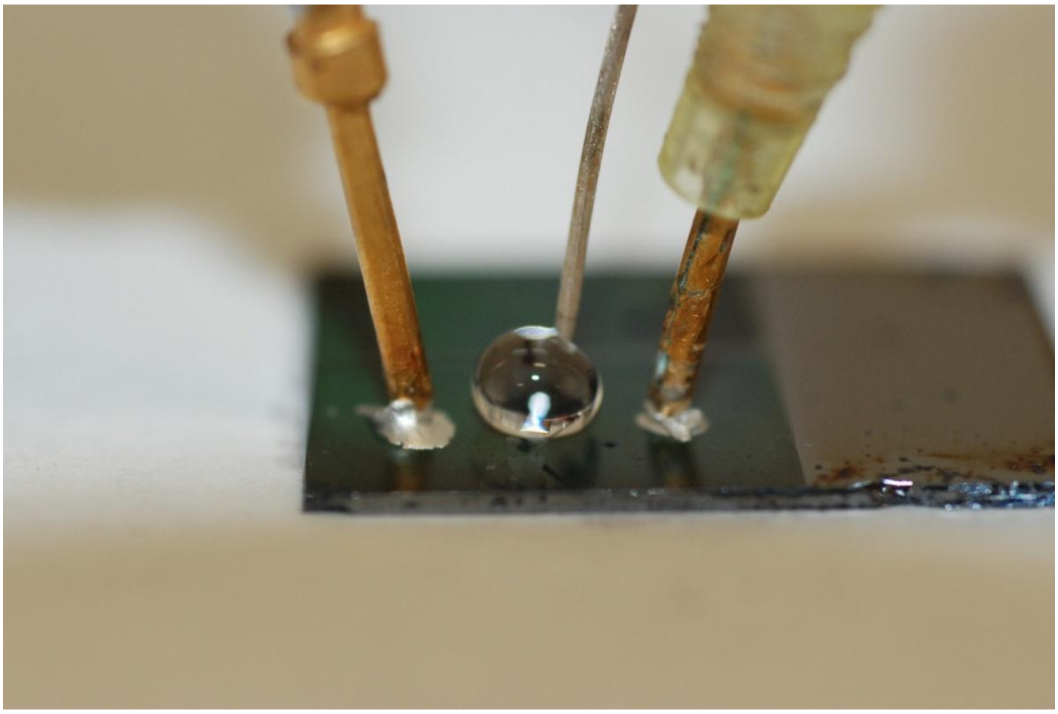

Figure S1.2

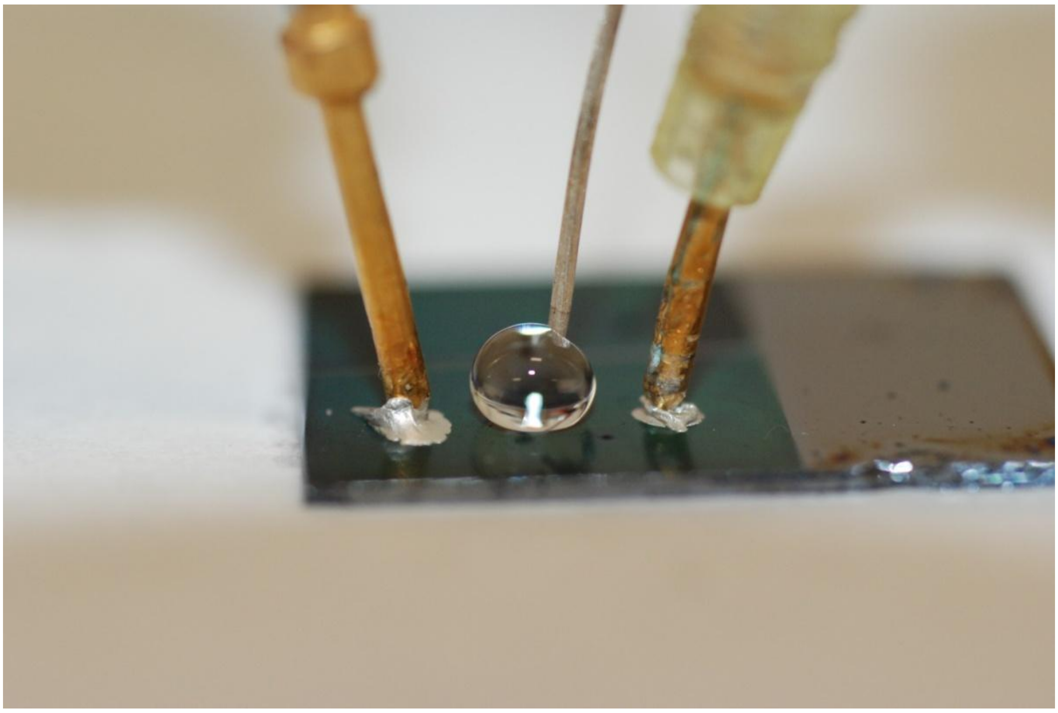

Figure S1.3

Supporting Information #2

In Figure S2.1, the response for different MgCl concentration, from 10^{-7} to 10^{-3} M, has been reported. The response of the device is expressed as the current modulation $\Delta I/I_0=|I_{ds,off}-I_{ds,0}|/I_{ds,0}$, where $I_{ds,off}$ is the off current ($V_{gs}>0$) and $I_{ds,0}$ the on current ($V_{gs}=0$). The reported values of

modulation depends on the MgCl concentration for different V_g. The red line, representing 10^{-7} M, produces a modulation in current that is lower for each V_g value respect to the green line of 10^{-5} M, and coherently 10^{-3} results with a higher response respect to 10^{-5} M.

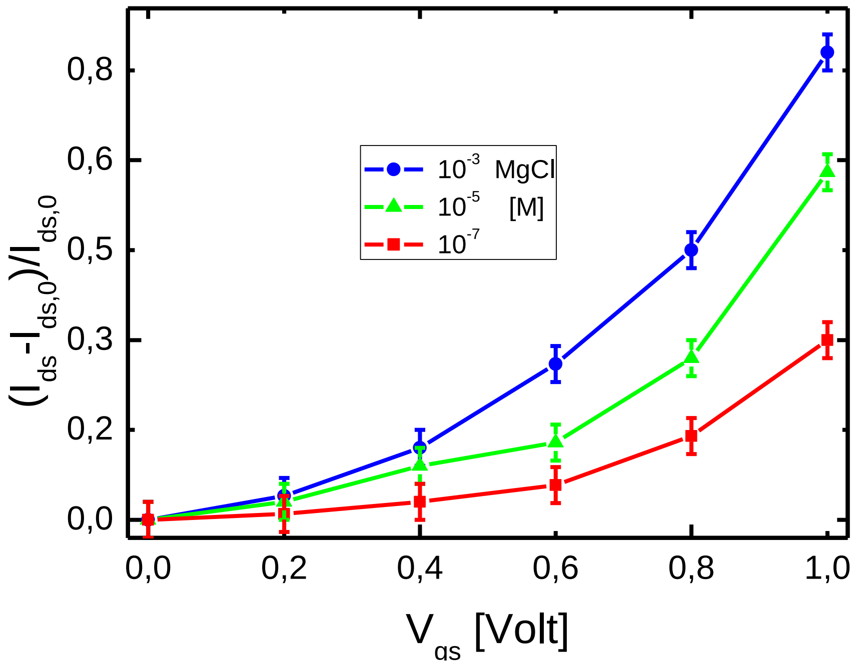

Figure S2.1
